# Evidence of autophagic vesicles in a patient with Lisch corneal dystrophy

**DOI:** 10.5935/0004-2749.20200027

**Published:** 2020

**Authors:** Arturo E. Grau, Sergio González, Pablo Zoroquiain, Pablo A. González, Daniela Khaliliyeh, Eugenia Morselli, Dennis Cortés

**Affiliations:** 1 Ophthalmology Department, Pontificia Universidad Católica de Chile, Santiago, Chile; 2 Pathology Department, Pontificia Universidad Católica de Chile, Santiago, Chile; 3 Molecular Genetics and Microbiology Department, ^Faculty of^ Biological Sciences, Pontificia Universidad Católica de Chile, Santiago, Chile; 4 Millennium Institute on Immunology and Immunotherapy, Faculty of Biological Sciences, Pontificia Universidad Católica de Chile, Santiago, Chile; 5 Physiology Department, Faculty of Biological Sciences, Pontificia Universidad Católica de Chile, Santiago, Chile

**Keywords:** Autophagy, Corneal dystrophies, hereditary, Corneal opacity, Vacuoles/pathology, Humans, Autofagia, Distrofias hereditárias da córnea, Opacidade da córnea, Vacúolos/patologia, Humanos

## Abstract

Lisch corneal dystrophy is a rare corneal disease characterized by the distinctive feature of highly vacuolated cells. Although this feature is important, the nature of these vacuoles within corneal cells remains unknown. Here, we sought to analyze corneal cells from a patient diagnosed with Lisch dystrophy to characterize the vacuoles within these cells. Analyses using histopathology examination, confocal microscopy, and transmission electron microscopy were all consistent with previous descriptions of Lisch cells. Importantly, the vacuoles within these cells appeared to be autophagosomes and autolysosomes, and could be stained with an anti-microtubule-associated protein 1A/1B-light chain 3 (LC3) antibody. Taken together, these findings indicate that the vacuoles we observed within superficial corneal cells of a patient with Lisch corneal dystrophy constituted autophagosomes and autolysosomes; this finding has not been previously reported and suggests a need for further analyses to define the role of autophagy in this ocular disease.

## Abstract (pt)

A distrofia corneana de Lisch é uma doença rara, caracterizada principalmente pela presença de células altamente vacuoladas. Embora esta característica seja importante, a natureza desses vacúolos dentro das células da córnea permanece des conhecida. Aqui, procuramos analisar as células da córnea de um paciente diagnosticado com distrofia de Lisch para caracterizar os vacúolos dentro dessas células. Análises utilizando exame histopatológico, microscopia confocal e microscopia eletrônica de transmissão foram todas consistentes com descrições previas de células de Lisch. Importante, os vacúolos dentro dessas células pareciam ser autofagossomos e autolisossomos, e poderiam ser corados com um anticorpo proteico 1A/1B-cadeia leve 3 (LC3) da proteína anti-microtúbulo associado a microtúbulos. Em conjunto, esses achados indicam que os vacúolos observados nas células superficiais da córnea de um paciente com distrofia corneana de Lisch constituíram autofagossomos e autolisossomos. Esse achado não foi relatado anteriormente e sugere a necessidade de mais análises para definir o papel da autofagia nessa doença ocular.

## INTRODUCTION

Corneal dystrophies are defined as inherited diseases that are typically bilateral, symmetric, slowly progressive, and unrelated to environmental or systemic factors^(1)^. However, exceptions exist for each of these typical characteristic features; moreover, corneal dystrophies can be subdivided based on the layer of the cornea primarily compromised^(1-3)^.

Lisch corneal dystrophy affects the corneal epithelium and is classified as a Category 2 dystrophy based on the IC3D classification: “a well-defined corneal dystrophy that has been mapped to one or more specific chromosomal loci (X-linked dominant pattern, locus Xp22.3), but the gene(s) associated with the onset of the disease remain(s) to be identified”^(2-4)^. By direct illumination, the epithelium typically displays localized gray opacities with different patterns; by indirect illumination, multiple densely crowded clear cysts can be identified^(3,5,6)^. The disease tends to be asymptomatic, but might manifest with blurred vision if the pupillary axis is involved^(3,5)^. Analysis of the epithelium by transmission electron microscopy (TEM) shows cells with a myriad of vacuoles that generally manifest in either of two forms: 1) vaguely flocculent or lamellar material with or without circumscribing membranes, or 2) more electron-dense whorled or membranous structures^(3,5)^. Thus far, the biological nature of these vacuoles has not been studied in detail.

Here, we report a case of Lisch corneal dystrophy in which histopathology analysis revealed the presence of autophagosomes and autolysosomes. These novel findings are discussed in detail.

## CASE REPORT

**Figure 1:**
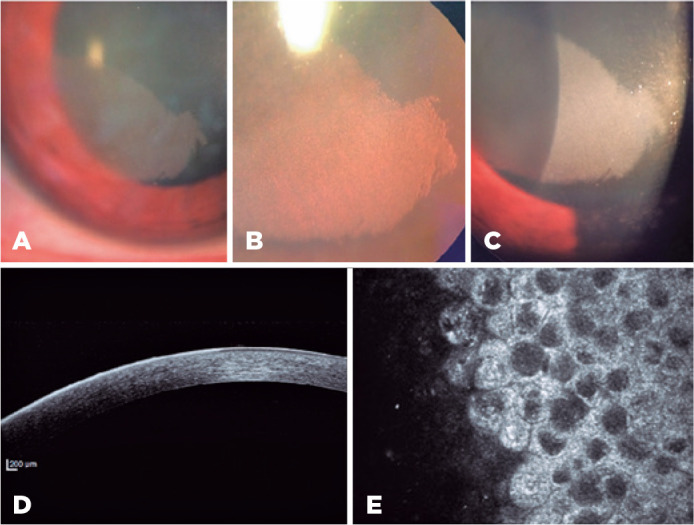
(A-C) Slit-lamp examination (SL) in the right eye showing a gray, banded shape, sharply demarcated corneal opacity with an intraepithelial dense microcystic aspect. (B) SL, retroillumination. (D) Anterior segment optical coherence tomography showing a hyperreflective epithelium in the affected region. (E) In vivo confocal microscopy showing abnormal epithelial cells with intracytoplasmic vacuoles, with highly hyperreflective cytoplasm and hyporeflective nuclei.

A 32-year-old woman with no relevant clinical history requested ophthalmologic evaluation after experiencing episodes of visual impairment in the right eye. Her best-corrected visual acuity was 20/25 in the right eye and 20/20 in the left eye. Slit-lamp analysis of the left cornea revealed no alterations. The right eye showed a gray, banded shape, as well as sharply demarcated corneal opacification with an intraepithelial dense microcystic aspect; this was more evident when observed with retroillumination due to the compromised visual axis (Figures 1A, B, and C). Anterior segment optical coherence tomography (Spectralis; Heidelberg Engineering, Heidelberg, Germany) showed hyperreflective epithelium in the affected cornea (Figure 1D). *In vivo* corneal confocal microscopy (Rostock Cornea Module, Heidelberg Engineering Retina Tomograph III, Heidelberg, Germany) showed abnormal epithelial cells with intracytoplasmic vacuoles, with highly hyperreflective cytoplasm and hyporeflective nuclei (Figure 1E).

## RESULTS

Importantly, we found in the eye of this patient morphological alterations consistent with Lisch corneal dystrophy, as previously described by Lisch et al.^(5)^. Hallmarks of Lisch corneal dystrophy were also confirmed by confocal microscopy. Consistent with the work of Kurbanyan et al.^(7)^, who recognized four characteristic features of abnormal epithelial cells in this dystrophy, we found: i) highly hyperreflective cytoplasm and hyporeflective nuclei, ii) uniform involvement of all epithelial layers within the affected areas, iii) well-defined borders with adjacent normal epithelium, and iv) involvement of the limbal area.

**Figure 2:**
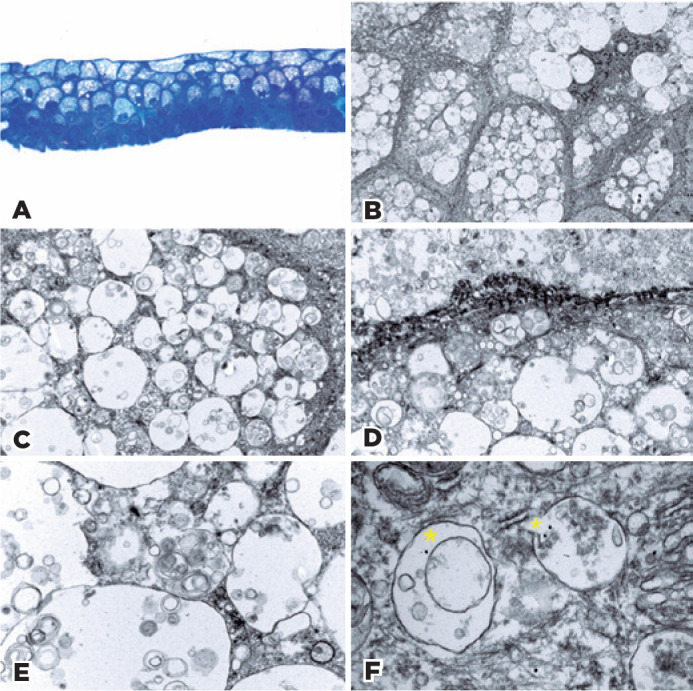
(A) Microscopy analysis of toluidine blue staining revealing scat- tered areas of vacuoles within basal and suprabasal cells. The vacuoles appear optically empty (400×). (B) Electron microscopy (EM) panoramic view showing vacuolated squamous epithelium. Vacuoles are larger and more abundant in superficial cells than in those of the basal layer. Uranyl acetate and lead citrate, original magnification 1,700×. C) EM, lead citrate and uranyl acetate staining, showing supranuclear vacuoles of different sizes along the entire thickness of the epithelium. Myelinoid bodies and debris are also present, especially at the deeper layers. These findings are consistent with autophagosome phenotype (6,000×). (D) EM, vacuoles of different sizes showing myelinoid structures. Uranyl acetate and lead citrate, original magnification 6,000×. (E) EM, details of vacuoles with multilayered bodies of thin electron-dense membranous and amorphous material, representing autophagic vacuoles. Uranyl acetate and lead citrate, original magnification 16,500×. (F) Immunoelectron microscopy showing gold particles, evidenced as a highly electron-dense band (yellow asterisks) concentrated in autophagic vacuole walls. Original magnification 43,000×.

To gain further insight into possible alterations in Lisch corneal dystrophy in this patient, a superficial keratectomy was performed. Histopathologic examination of the epithelium revealed keratinocytes with round bland nuclei and a conserved nucleus to cytoplasm ratio (Figure 2A). The cytoplasm showed scattered areas of optically empty vacuoles within suprabasal cells. Cells in the upper layers exhibited no maturation anomalies. No dyskeratosis, parakeratosis, subepithelial material, or inflammation were observed (Figure 2A). Additional analysis by TEM showed vacuoles of different sizes within the entire thickness of the epithelium; these were more numerous and larger in the superficial layers (Figures 2B and C). In the vacuoles, whorled structures consistent with myelinoid bodies and debris were observed, especially in the deeper layers (Figures 2D and 2E). Morphologically, these optical microscopy and electron microscopy findings were similar to those described by Wessel et al.^(8)^. To confirm the nature of the observed structures, immunostaining of the TEM slides was performed with an anti-microtubule-associated protein 1A/1B-light chain 3 (LC3) gold-labeled antibody, as LC3 is a key protein required for the formation of autophagosomes and autolysosomes. In basal conditions, LC3-I is diffuse in the cell; when autophagy is induced, LC3-I is cleaved by an autophagy-related (ATG) protein, ATG4, thus forming LC3-II, which is inserted in the autophagosome membrane^(9)^. Importantly, all vacuoles within cells in the superficial layers of corneal tissue from our patient expressed LC3, which is normally localized in the membrane of autophagic vesicles (Figure 2F).

## DISCUSSION

Autophagy is a lysosome-mediated degradation process of non-essential and damaged cellular constituents, which constitutes a mechanism to preserve cellular homeostasis by maintaining the balance between organelle biogenesis, protein synthesis, and clearance of these cellular components^(10)^. Importantly, alterations in this proteolytic process may result in the intracellular accumulation and retention of misfolded proteins or defective organelles, causing different pathological conditions. Furthermore, defects in autophagy have been recognized in granular corneal type 2 dystrophy^(11)^.

In conclusion, we found that vacuoles present within superficial corneal cells of a patient with Lisch corneal dystrophy contained autophagosomes and autolysosomes, a novel finding that has not been previously reported. Given these results, we hypothesize that autophagy could promote the occurrence of this disease or that its manifestation may result as a consequence of other altered events associated with this condition. These findings should be confirmed in additional patients to validate our hypothesis.
